# Editorial: Multiple sclerosis and related disorders: challenges and approaches to mechanisms, biomarkers, and therapeutic targets

**DOI:** 10.3389/fneur.2024.1427299

**Published:** 2024-05-17

**Authors:** Yang Zhou, Philippe Patrick Monnier, Jin-Zhou Feng, Shou-Gang Guo, Cong-Cong Wang

**Affiliations:** ^1^Department of Neurology, The First Affiliated Hospital of Shandong First Medical University, Shandong Provincial Qianfoshan Hospital, Jinan, China; ^2^Department of Physiology, University of Toronto, Toronto, ON, Canada; ^3^Department of Neurology, The First Affliated Hospital of Chongqing Medical University, Chongqing, China; ^4^Department of Neurology, Shandong Provincial Hospital Affiliated to Shandong First Medical University, Shandong Academy of Medical Sciences, Jinan, China; ^5^Shandong Institute of Neuroimmunology, Jinan, China

**Keywords:** MS, NMOSD, MOGAD, molecular mechanisms, biomarkers, therapeutic

Multiple sclerosis (MS)-related disorders are chronic autoimmune disease of the central nervous system (CNS) characterized by inflammation, demyelination, and neurodegeneration. Common MS-related disorders include MS, neuromyelitis optica spectrum disorder (NMOSD), myelin oligodendrocyte glycoprotein-antibody-associated disorders (MOGAD), and other diseases, which have relatively independent clinical features and diagnostic markers. In addition, biomarkers of MS-related disorders are employed in clinical diagnosis, estimation of disease risk or prognosis, assessment of disease staging, and monitoring of disease progression or response to therapy (1). However, at present, there is no cure for MS-related disorders. Disease-modifying therapies (DMTs) represent the mainstay of treatment, and patients are generally required to undergo lifelong treatment. In order to facilitate the dissemination of the latest research findings in this field, we have organized this Research Topic. The Research Topic comprises 10 manuscripts that expand contemporary knowledge and understanding of the mechanisms, biomarkers, and therapeutic targets of MS and related diseases.

MS is a CNS inflammatory demyelinating disease that involves white matter. The pathogenesis of MS is mainly due to auto-reactive lymphocytes (T and B cells), innate immune and microglial cells, which synergistically mediate myelin loss, secondary axonal injury, and astrocyte reactive hyperplasia (2). The disease may be related to genetic, environmental, viral infection, and other factors. Neurofilament light (NfL) has been identified as a marker of axonal damage. Mi et al. conducted a study in which they analyzed NfL levels in the plasma of MS patients in conjunction with clinical and magnetic resonance imaging (MRI) assessments. The level of plasma NfL is correlated with the activity and severity of MS, and is therefore anticipated to become a novel biomarker for the assessment of MS activity and disease status. The protein growth arrest specific 6 (Gas6) and its tyrosine kinase receptors Tyro-3, Axl, Mer (TAMs) have been linked to the remyelination of neurons and the stimulation of oligodendrocyte survival. D'Onghia et al. assessed the soluble levels of Gas6-TAMs in serum and cerebrospinal fluid (CSF), at the time of MS diagnosis, and to evaluate their possible correlations with short-term disease severity. The serum levels of Axl were found to be higher in patients with lower disability at the time of onset, while the serum levels of Gas6 were higher in patients with lower disability over time. These findings suggest that serum Gas6 may be a reliable prognostic biomarker. Zhang X. et al. reported that the nationwide prevalence of CSF-OCB in Chinese MS patients was 76.4%, and demonstrated that their diagnostic approach is effective in differentiating MS from other CNS diseases. The prevalence of CSF-OCB demonstrated an association with high latitude and altitude in Chinese MS patients. Dorsch et al. conducted a study to examine disease progression in patients with MS, defined using an objective, home-based assessment of motor functions, compared to 3-month confirmed disease progression (3-mCDP) as defined by the EDSS. It may be beneficial to reduce the length of observation periods during clinical trials, which would enhance confidence in the ability to identify progression events associated with MS. Zhang L. et al. proposed that lacune may serve as a potential MRI biomarker in MS. In this study, they sought to elucidate the relationship between small vessel disease (CSVD) and MS using lacune as a biomarker. Arisi et al. identified drug-dependent alterations in miRNA profiles in patients with relapsing-remitting MS (RRMS) and proposed a series of candidate miRNAs that they believe may be involved in the corresponding pharmacological mechanisms.

NMOSD is a rare relapsing neuroinflammatory autoimmune disease that primarily affects the optic nerves and spinal cord. Most cases exhibit aquaporin-4-antibody positivity. The pathological mechanism of NMOSD differs from that of MS, primarily involving autoimmune injury of astrocytes, secondary demyelinating changes, and perivascular inflammation, including neutrophil and eosinophilic infiltration (3). The most common associations of MOGAD include central nervous system demyelination, which manifests as acute disseminated encephalomyelitis in children, optic neuritis (ON) and transverse myelitis (TM) in children and adults. Unlike MS, MOGAD does not typically present with radiographic white matter changes (4). Wang et al. demonstrated that an elevated systemic immune-inflammation index (SIRI) may serve as a distinguishing indicator for differentiating MOGAD from AQP4-IgG-positive NMOSD. Additionally, they observed that decreased MLR levels may be associated with an increased probability of MOGAD recurrence. Ma et al. demonstrated that patients with mild disability NMOSD exhibited compensatory increases in local network properties to maintain stability at the system level. Additionally, they observed that alterations in the morphological network nodal properties of NMOSD patients were more relevant for clinical assessments when compared with functional network nodal properties. Furthermore, they found that these alterations exhibited predictive values of worsening in the Expanded Disability Status Scale (EDSS) scores. Asseyer et al. found that lower cervical spinal cord volume was associated with increased pain in patients with AQP4-IgG-positive NMOSD. Furthermore, regional spinal cord MRI measures have been identified as being crucial for monitoring disease-related changes within the spinal cord of individuals diagnosed with AQP4-IgG-positive NMOSD and MOGAD.

Finally, autoimmune encephalitis is defined as a non-infectious, immune-mediated inflammatory process that affects the brain parenchyma. It is characterized by the presence of neural antibodies in a significant proportion of patients (5). Anti-NMDA receptor (NMDAR) encephalitis is a prevalent autoimmune encephalitis, with GluN1 antibodies as a key causal factor. Prompt identification is of critical consequence. Iizuka et al. demonstrated that the severity of the disease in patients and the presence of four key symptoms were associated with higher levels of GluN1-ab antibodies in CSF samples taken at the time the disease was first diagnosed. The results may indicate a potential link between the presence of these antibodies and the subsequent one-year functional status of patients.

In conclusion, the articles in this Research Topic expand current knowledge regarding the mechanisms, biomarkers, and therapeutic targets of MS and related diseases. The findings of these studies offer novel scientific evidence and provide insights into the latest advances in this rapidly developing field.

## Author contributions

YZ: Writing – original draft. PM: Writing – review & editing. J-ZF: Writing – review & editing. S-GG: Writing – review & editing. C-CW: Writing – review & editing.
